# Modeling spinocerebellar ataxias 2 and 3 with iPSCs reveals a role for glutamate in disease pathology

**DOI:** 10.1038/s41598-018-37774-2

**Published:** 2019-02-04

**Authors:** Ching-Yu Chuang, Chih-Chao Yang, Bing-Wen Soong, Chun-Ying Yu, Shu-Hwa Chen, Hsiang-Po Huang, Hung-Chih Kuo

**Affiliations:** 1 Genomics Research Center, Academia Sinica, Taipei, Taiwan; 2 Institute of Cellular and Organismic Biology, Academia Sinica, Taipei, Taiwan; 3 Departments of Neurology, National Taiwan University Hospital, Taipei, Taiwan; 4 Departments of Neurology, National Yang-Ming University Faculty of Medicine and Taipei Veterans General Hospital, Taipei, Taiwan; 5 Lab of Systems and Network Biology, Institute of Information Science, Academia Sinica, Taipei, Taiwan; 6 Graduate Institute of Medical Genomics and Proteomics, College of Medicine, National Taiwan University, Taipei, Taiwan; 7 Graduate Institute of Clinical Medicine, Taipei Medical University, Taipei, Taiwan

## Abstract

Spinocerebellar ataxias 2 and 3 (SCA2 and SCA3) are dominantly inherited neurodegenerative diseases caused by expansion of polyglutamine-encoding CAG repeats in the affected genes. The etiology of these disorders is known to involve widespread loss of neuronal cells in the cerebellum, however, the mechanisms that contribute to cell death are still elusive. Here we established SCA2 and SCA3 induced pluripotent stem cells (iPSCs) and demonstrated that SCA-associated pathological features can be recapitulated in SCA-iPSC-derived neurons. Importantly, our results also revealed that glutamate stimulation promotes the development of disease-related phenotypes in SCA-iPSC-derived neurons, including altered composition of glutamatergic receptors, destabilized intracellular calcium, and eventual cell death. Furthermore, anti-glutamate drugs and calcium stabilizer treatment protected the SCA-iPSC-derived neurons and reduced cell death. Collectively, our study demonstrates that the SCA-iPSC-derived neurons can recapitulate SCA-associated pathological features, providing a valuable tool to explore SCA pathogenic mechanisms and screen drugs to identify potential SCA therapeutics.

## Introduction

Spinocerebellar ataxia (SCA) comprises a group of genetic neurological disorders that display clinical features including gait ataxia, cerebellar dysarthria, ophthalmoplegia, pyramidal or extrapyramidal signs, and peripheral neuropathy^1,2^. To date, approximately seven SCA subtypes have been described as polyglutamine (polyQ) disorders. These include the more prevalent SCA1, SCA2, SCA3 and SCA6, along with less prevalent SCA7, SCA17 and dentatorubropallidoluysian atrophy. PolyQ disorders are caused by an abnormal expansion of trinucleotide CAG repeats in the translated region of their respective genes^3^. Although the affected genes in various types of SCAs have disparate functions, several pathophysiological characteristics, such as mitochondrial defects, transcriptional dysregulation, protein aggregation, ion channel defects, dysregulated autophagy, and neuronal cell death are common among SCA subtypes^3,4^.

SCA2 and SCA3 are two of the most common SCA subtypes^3^, and as such, they have been the most widely studied. Much has been learned about potential underlying mechanisms of SCA pathology from transgenic expression of orthologous genes with expanded polyQ in model organisms. For example, nuclear inclusion formation and late-onset neurodegeneration caused by Q78 protein expression has been described in a Drosophila SCA3 model^5^. Moreover, the relationship between disease severity and CAG-repeat length has been demonstrated in a SCA3 transgenic mice model carrying a single or multiple copies of Q64-84^6^. Additionally, neuronal dysfunction and Purkinje cell loss were shown to be unnecessary for the formation of intranuclear aggregates in SCA2-58Q transgenic mice^7^. However, it is still unclear whether animal models of SCA can faithfully recapitulate clinical features of SCA, as there are substantial genetic and anatomical differences between these models and human patients.

Currently, there is no effective treatment to prevent disease progression or alleviate SCA symptoms. In order to conquer these devastating diseases, it will be essential to establish human-derived SCA disease models to study mechanisms and screen drugs for treatment. The recent success in pluripotency reprogramming technology enables us to derive disease-specific induced pluripotent stem cells (iPSCs) from patients. Because of their pluripotent character, these cells can be differentiated into many cell types, including neurons, and have emerged as an important tool to explore the pathological progression of neurodegenerative diseases *in vitro*. Thus, iPSCs can provide a source for disease-relevant cell types that are normally difficult to access. Currently, human iPSC derived neural cells are commonly used to model neurodegenerative diseases, such as amyotrophic lateral sclerosis (ALS), spinal muscular atrophy, Parkinson’s disease, Alzheimer’s disease (AD) and Huntington’s disease (HD)^8^. In contrast, iPSC-based disease modeling and drug testing for SCA2 and SCA3 have only been described in a limited number of studies^9–11^. For example, Koch *et al*. first demonstrated that ataxin-3 aggregates that developed in glutamate-treated SCA3-iPSC-derived neurons were initiated by Ca^2+^-dependent calpain-mediated proteolysis^9^.

In the present study, we generated multiple iPSC lines from both SCA2 and SCA3 patients and demonstrated that the hallmark pathological features of SCA2 and SCA3, including neuronal cell death and polyQ aggregation, can be recapitulated in the SCA-iPSC-derived neuronal cell population. Furthermore, our results revealed that long-term glutamate treatment worsened the pathological phenotypes, downregulated the component genes of glutamate receptor, compromised intracellular calcium stability, and promoted neuronal degeneration in the SCA-iPSC-derived neurons. Moreover, our results demonstrated that anti-glutamate drugs were able to ameliorate the pathological phenotypes that we discovered in SCA2- and SCA3-iPSC-derived neurons. Thus, our studies support the notion that glutamate-associated pathways contribute significantly to the pathological phenotypes of SCA-iPSC-derived neurons and that these pathways can serve as a drug targets for developing potential SCA therapeutics.

## Results

### Generation and characterization of SCA2- and 3-iPSCs

**Figure 1:**
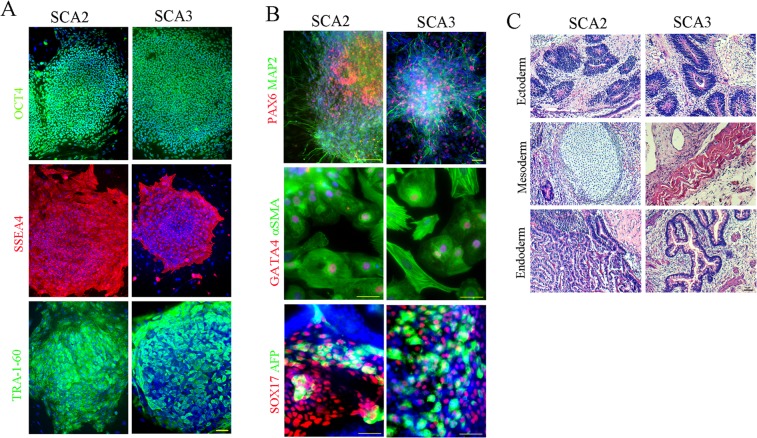
Characterization of representative SCA2-1 (iSCA2-17) and SCA3-1 (iSCA3-1) iPSCs. Immunostaining analysis for (**A**) pluripotency-associated markers in representative SCA-iPSC colonies and (**B**) three embryonic germ layer-associated markers in differentiated SCA-iPSC derivatives. (**C**) Hematoxylin and eosin staining of teratomas derived from representative SCA-iPSCs. All scale bars: 50 μm.

**Figure 2:**
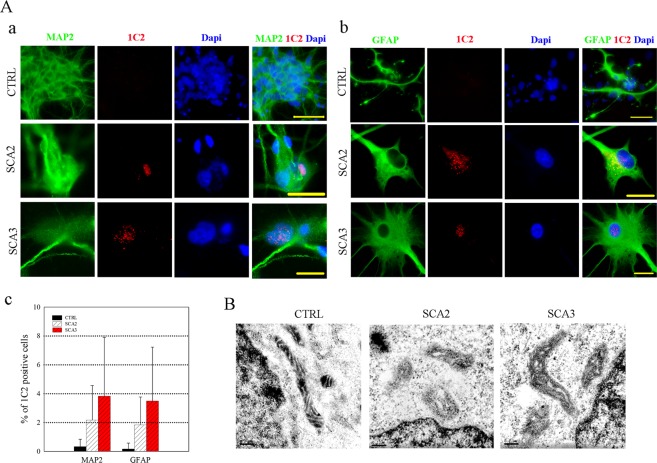
Recapitulating SCA-associated disease phenotypes in the SCA-iPSC-derived neural cells. (**A**) Intracellular polyQ accumulation occurs in (a) neurons (MAP2^+^) and (b) glial cells (GFAP^+^). Detection of polyQ aggregates was achieved using the anti-PolyQ-Expansion Disease Marker 1C2 antibody (red). Scale bar: 20 μm. (c) Quantitative analysis of polyQ aggregates in neurons and glial cells. (**B**) EM analysis of ultrastructural features in SCA-iPSC-derived neurons. Scale bar: 200 nm. CTRL-1 & 2, SCA2-1 & 2 and SCA3-1 & 2 iPSC lines were used in the experiments.

To generate SCA-iPSCs and control iPSCs, peripheral blood mononuclear cells (PBMCs) or dermal fibroblasts, obtained from four patients with SCA2 or SCA3, and three normal individuals, were transduced with Sendai or retro viruses, encoding OCT4, SOX2, KLF4 and c-MYC (OSKM) (Table S1). Cell clusters resembling human embryonic stem cell-like colonies started to emerge approximately 20 to 30 d post-viral infection (Fig. S1A). For each case, we selected 10 to 40 iPSC clones for further expansion and characterization. PCR and RT-PCR analysis confirmed the absence of exogenous OSKM, Sendai viral (SeV) vector expression, and reactivation of endogenous pluripotency-associated genes in the SCA- and control-iPSC lines (Fig. S2). Further, IF staining revealed that all the tested iPSC lines expressed pluripotency-associated markers such as OCT4, SSEA4, TRA-1-60 (Figs 1A and S1B). To assess their pluripotent characteristics, the iPSCs were tested by *in vitro* differentiation via EB formation, and *in vivo* teratoma formation assays. IF analysis showed that the SCA-iPSCs were able to differentiate into cell types expressing markers of all three embryonic germ layers under *in vitro* differentiation conditions (Figs 1B and S1C). After intramuscular injection of undifferentiated SCA-iPSCs into immunocompromised mice, teratomas consisting of cell types representing all three embryonic germ layers were formed (Figs 1C and S1D). All the SCA-iPSC lines also showed normal chromosomal karyotypes (Fig. S1E). Furthermore, combined PCR and genomic DNA sequencing analysis confirmed that expanded CAG repeats were present in *ATXN2* and *ATXN3* in SCA2- and SCA3-iPSCs, respectively (Fig. S3 and Table S1). We also demonstrated that the SCA-iPSC are able to give rise to highly-enriched neuronal populations with more than 70% of the cells expressing neuronal markers, TUJ1 or MAP2 (Figs 2A and S4). Although our neural differentiation protocol was not designed to obtain a pure cerebellar neuronal population, some of the neurons in the mixed population expressed granular cell precursor markers, such as ZIC1, ZIC2, ZIC3, and ATH1, as well as Purkinje cell markers GAD67, LHX5 and CALB1 (Fig. S4). Together, these results demonstrate that iPSC with robust *in vitro* differentiation potentials can be reprogrammed from the somatic cells of SCA2 and SCA3 patients.

### SCA2- and SCA3-iPSC-derived neurons exhibit SCA pathological phenotypes

As polyQ aggregation is a hallmark feature of SCA2 and SCA3, it was of interest to know whether SCA-iPSC derived neurons can recapitulate this phenotype under regular neural culture conditions. To this end, antibody 1C2 (specific for polyQ tracts) was used in conjunction with MAP2 (neuronal marker) or GFAP (glial cell marker) to investigate the pattern of polyQ aggregation in SCA-iPSC-derived neurons (Fig. 2A). IF staining of the SCA-iPSC-derived neurons revealed that punctate 1C2 signal can be readily detected in the nucleus and occasionally in the cytoplasm of SCA-iPSC-derived neurons or glial cells, whereas no staining was detected in the CTRL-iPSCs (Fig. 2Aa,b). Quantifying the 1C2 positive cells, however, revealed that the percentages of 1C2-stained cells were low and variable in both SCA-iPSC-derived neurons and glia (Fig. 2Ac). Notably, electron microscopy analysis also revealed distorted mitochondrial microstructures, such as lost or undefined cristae, swollen matrix, or pale staining of matrix, in the SCA2 or SCA3-iPSC-derived neurons (Fig. 2B). Together, these results demonstrate that SCA-iPSC-derived neurons exhibit SCA-associated pathological phenotypes *in vitro*.

### Glutamate receptor signaling is affected in SCA-iPSC-derived neuronal populations cultured in glutamate-containing medium

**Figure 3:**
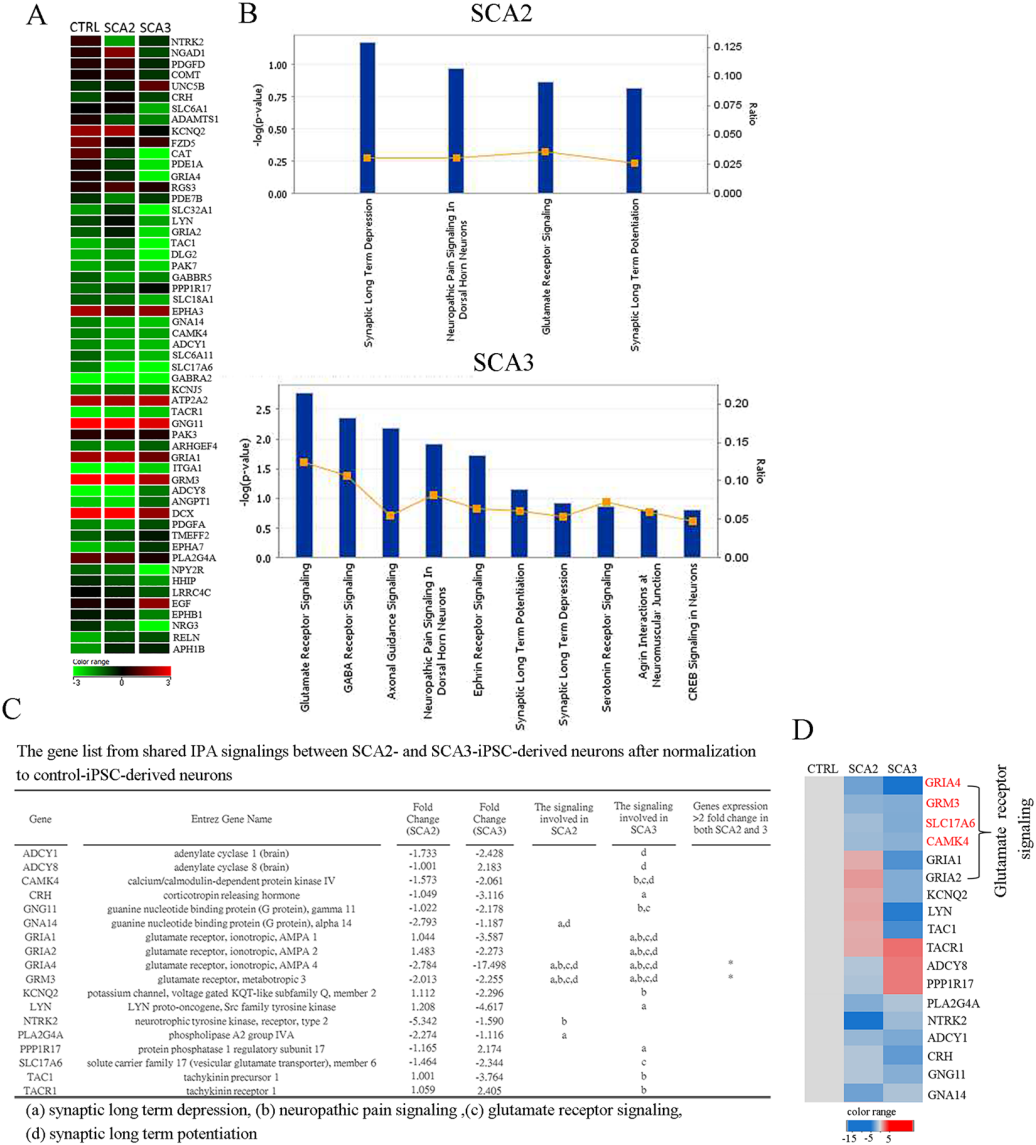
Transcriptome profiling revealed that genes associated with glutamate signaling were affected in SCA-iPSC-derived neuronal populations. (**A**) The heatmap shows fold changes of genes included in the gene ontology (GO) term, ‘neurotransmitters and other nervous system signaling’. Results from CTRL-, SCA2- and SCA3-derived neurons are shown. (**B**) The list of signaling categories predicted by IPA analysis of microarray results. The analysis was performed using data with gene expression > 2 fold between CTRL-iPSC-derived neuronal populations, and SCA2- or SCA3-iPSC-derived neurons. The x-axis represents the pathway. The y-axis for the line and square symbols is the ratio of the number of genes from the dataset that map to the pathway and the number of all known genes ascribed to the pathway. The y-axis for the bars is based on Fisher’s exact test *p*-value. The score cutoff was selected by a −log (*p*-value) > 0.8. (**C**) The gene list and (**D**) heatmap analysis of shared signaling differences between CTRL-iPSC-derived neurons and SCA-iPSC-derived neuronal populations. The fold changes of genes are shown as upregulated or downregulated in SCA-iPSC-derived neurons after normalization to CTRL-iPSC-derived neuronal populations. CTRL-1, 2 & 3, SCA2-1 & 2 and SCA3-1 & 2 iPSC lines were used in the experiments.

In order to further explore pathological features of SCA-iPSC-derived neurons, we compared the global gene expression profiles of CTRL- and SCA-iPSC-derived neurons cultured in conventional neural culture conditions, containing 100 µM glutamate from the non-essential amino acids (NEAA) supplement. Genes with at least two-fold change of expression between normal control and SCA-iPSC-derived neurons were identified, and a heatmap showed decreases in several genes under the gene ontology (GO) term of “Neurotransmitters and Other Nervous System Signaling” (Fig. 3A). To further characterize gene expression differences, Ingenuity Pathway Analysis (IPA) software was used to reveal that pathways involved in synaptic long-term depression, neuropathic pain signaling, glutamate receptor signaling and synaptic long-term potentiation were aberrantly regulated in both SCA2- and SCA3-iPSC-derived neurons (Fig. 3B). Among these pathways, glutamate receptor signaling was most significantly affected, especially in SCA3 cells (Fig. 3B,C). Moreover, we found several glutamate receptor-related genes, including GRIA4 (glutamate ionotropic receptor AMPA type subunit 4) and GRM3 (metabotropic glutamate receptor 3) were downregulated in SCA2-iPSC-derived neurons. Similarly, in SCA3-iPSC-derived neurons, CAMK4 (calcium/calmodulin-dependent protein kinase IV), GNC11 (guanine nucleotide binding protein gamma 11), GRIA1(glutamate ionotropic receptor AMPA type subunit 1), GRIA2 (glutamate ionotropic receptor AMPA type subunit 2), GRIA4, GRM3 and SLC17A6 (solute carrier family 17, member 6) were significantly downregulated (Fig. 3C,D). Together, these data suggest that glutamate-associated pathways may be preferentially affected in SCA-iPSC-derived neuronal populations.

### Glutamate treatment alters the expression of glutamate receptor-related genes in SCA-iPSC-derived neurons

**Figure 4:**
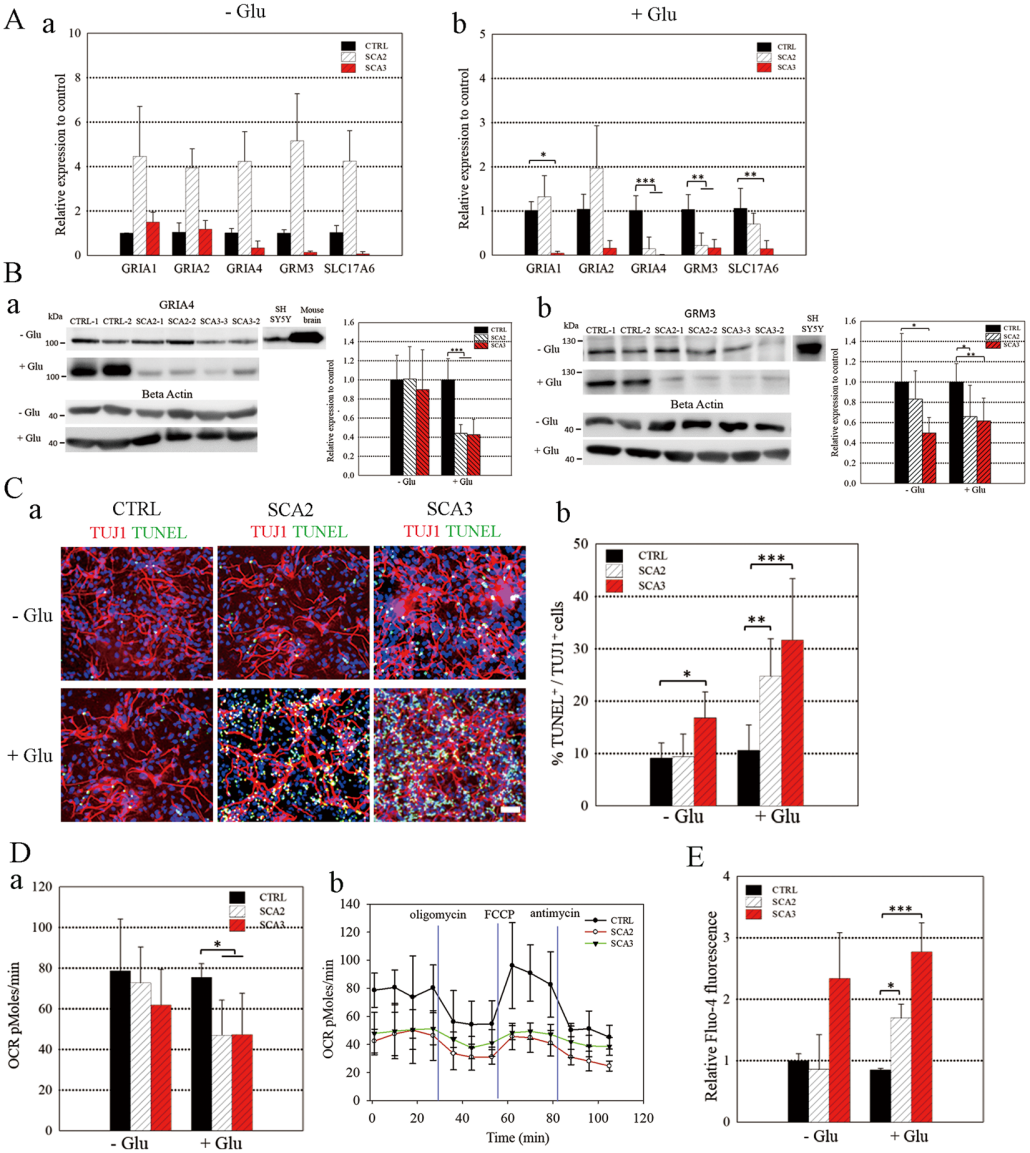
Culturing with glutamate enhanced expression differences of glutamate receptor-related genes and SCA-associated pathological phenotypes in the SCA-iPSC-derived neuronal populations. (**A**) RT-qPCR analysis of selected glutamate signaling-associated genes in control, SCA2- and SCA3-iPSC-derived neurons cultured (a) without or (b) with glutamate. Relative gene expression was first normalized to GAPDH, and then calculated as the fold change relative to control cells. (**B**) Immunoblotting and quantitative analysis of protein expression for (a) GRIA4 and (b) GRM3 in SCA-iPSC-derived neuronal populations without or with glutamate treatment. Relative protein expression was first normalized to β-ACTIN, and then fold changes compared to control were calculated. The full-length blots are presented in Fig. S10. (**C**) SCA-iPSC-derived neuronal cultures treated with or without glutamate were assessed for apoptosis with a TUNEL assay. (a) Neurons were stained with TUJ1 (red) and TUNEL positive signals are green. Nuclei were stained with DAPI. Scale bar: 50 μm. (b) Quantitative analysis of TUNEL^+^ cells among TUJ1^+^ neuronal population. At least 1 × 10^4^ cells were counted for each experiment. (**D**) Measurement of oxygen consumption rates (OCR) by the Seahorse XF24 Extracellular Flux Analyzer. (a) Comparison of basal respiration among SCA2-, SCA3- and CTRL-iPSC-derived neuronal populations treated with or without glutamate. (b) The mitochondrial stress test in neurons cultured in glutamate-containing media. Vertical lines (Blue) indicate the time points at which different treatments were administered. (**E**) Intracellular calcium measurement by Fluo-4 fluorescence intensity in SCA-iPSC-derived neuronal populations without or with glutamate. All data represent the mean ± SD. ***P < 0.001, **P < 0.01, *P < 0.05. −Glu: without glutamate. +Glu: with glutamate. CTRL-1 & 2, SCA2-1 & 2 and SCA3-1 & 2 iPSC lines were used in the experiments.

To confirm our microarray and IPA results, we next set out to determine whether the presence of glutamate in the culture medium was associated with alterations in glutamate receptor gene expression. In order to do so, we used RT-qPCR to compare the expression levels of the aforementioned glutamate receptor genes (Fig. 3D) between iPSC-derived neurons cultured in medium without or with glutamate (100 µM) (Fig. 4A). We found that in the absence of glutamate, the expression levels of several glutamate receptor-related genes, such as GRIA4, GRM3 and SLC17A6, were all lower in SCA3-iPSC-derived neurons compared to CTRL-iPSC-derived neurons, although no statistically significant differences were found (Fig. 4Aa). Furthermore, in line with our microarray results, glutamate treatment further reduced the expression of glutamate receptor-related genes, including GRIA1, GRIA2, GRIA4, GRM3 and SLC17A6, in the SCA-iPSC-derived neurons compared to their counterparts derived from CTRL-iPSCs (Fig. 4Ab). Western blot analysis also demonstrated that the protein levels of GRIA4 and GRM3 were significantly reduced in SCA-iPSC-derived neurons cultured in glutamate-containing medium, while the neuronal marker (TUJ1) was unaffected (Figs 4Ba,Bb and S5). Thus, these results suggest that glutamate treatment has a profound impact on the expression of glutamate receptor-related genes.

### Glutamate treatment promotes the development of SCA-associated pathological phenotypes in SCA-iPSC-derived neurons

Since the above results suggested glutamate receptor-associated signaling was greatly affected in SCA-iPSC-derived neurons, we hypothesized that glutamate may contribute to the development of SCA pathological phenotypes in the SCA-iPSC-derived neurons. To test this hypothesis, we used various assays to investigate the impact of glutamate on cell death and mitochondrial dysfunction on the iPSC-derived neurons. To determine the dose and temporal effects of glutamate on SCA-neurons, we cultured iPSC-derived neurons in medium supplemented with different concentrations (50 and 100 μM) of glutamate for various time periods (15, 30 and 60 days). The TUNEL assay revealed that under conditions of 0 or 50 μM glutamate, overt cell death in CTRL and SCA2-iPSC-derived neurons was not observed (Fig. S6). However, a higher dose of glutamate (100 μM) increased cell death in both SCA2- and SCA3-iPSC-derived neurons in a time-dependent manner, particularly after 60 days of glutamate supplementation. These results suggest that long-term high dose glutamate treatment has a detrimental effect on the SCA-iPSC-derived neurons.

By analyzing TUNEL-positive signals in TUJ1^+^ neurons, we found that CTRL- and SCA2-iPSC-derived neurons had similar percentages of apoptotic cells under glutamate-free conditions, but SCA3-iPSC-derived neurons showed increased cell death compared to CTRL (Fig. 4C). After addition of glutamate (100 μM, 60 days), increased cell death was found in both SCA2- and SCA3-iPSC-derived neurons compared to CTRL-iPSC-derived neurons. Furthermore, glutamate did not cause obvious cell death in the CTRL-iPSC-derived neurons (Figs 4C and S7A).

To explore the effect of glutamate on the mitochondrial function in SCA-iPSC-derived neurons, oxygen consumption rate (OCR) was measured by the Seahorse XF-24 metabolic flux analyzer to determine cellular respiration (Fig. 4D). Our analysis demonstrated that glutamate treatment significantly reduced the basal OCR in both SCA2- and SCA3-iPSCs-derived neurons compared to controls (Fig. 4Da). However, glutamate treatment had no overt effect on the neurons derived from CTRL-iPSCs (Fig. S7B). We also carried out a mitochondrial stress test to further test the effect of glutamate on mitochondrial function in SCA-iPSC-derived neurons. To this end, we sequentially treated the iPSC-derived neurons with three stress inducers, Oligomycin (ATP synthase inhibitor), FCCP (mitochondrial uncoupler) and Antimycin (complex III inhibitor). Accordingly, the Seahorse analysis showed that the OCR response to stress inducers was as expected in CTRL-iPSCs-derived neurons cultured in glutamate-containing medium (decreased after Oligomycin treatment, indicating ATP-linked respiration and proton leak; increased after FCCP treatment, indicating the maximal respiration capacity; decreased after Antimycin treatment, which shuts down mitochondrial respiration, indicating non-respiratory oxidation). On the other hand, SCA-iPSC-derived neurons showed blunted responses to the sequential stressor treatment (Fig. 4Db), suggesting that long term glutamate exposure might damage mitochondrial bioenergetic function, leading to cell death in the SCA-iPSC-derived neuronal populations.

Since it has been reported that dysregulation of calcium homeostasis can activate sequential toxic signaling cascades that lead to neuronal death^12^, it was of interest to know whether the pathological events observed in the SCA-iPSC-derived neurons were associated with increased calcium flux resulting from altered glutamate signaling. By measuring the fluorescence intensity of the calcium indicator dye, Fluo-4, our results showed the calcium level in the SCA-iPSC-derived neurons was significantly higher than CTRL-iPSC-derived neurons in glutamate-containing culture medium (Fig. 4E). In light of the gene expression data, these results suggest that glutamate receptor signaling is likely to increase calcium flux and disturb mitochondrial function in SCA2 and SCA3 neurons, thereby playing an important role in regulating neuronal cell death in multiple SCA subtypes.

### Anti-glutamate drugs mitigate the pathological phenotypes of SCA2- and SCA3-iPSC-derived neurons

**Figure 5:**
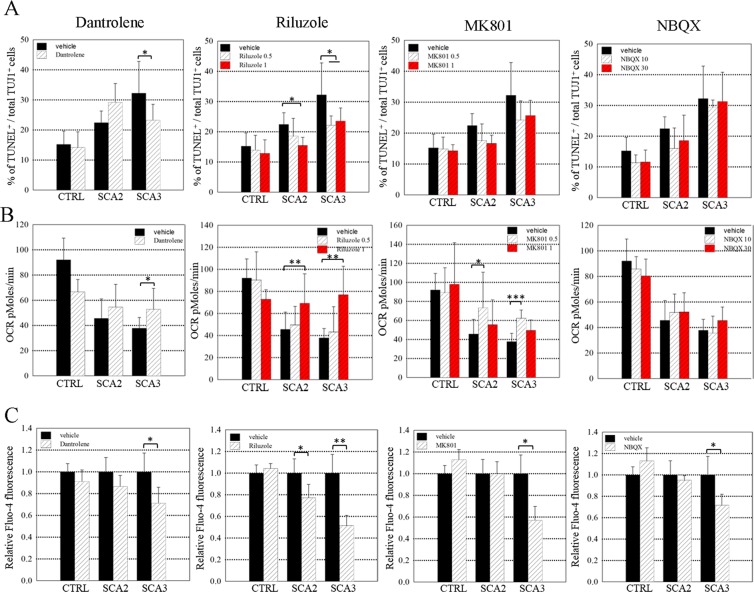
A calcium stabilizer and anti-glutamate drugs alleviated the pathological phenotypes of SCA-iPSC-derived neuronal populations. SCA-iPSC-derived neurons were treated with Dantrolene, Riluzole, MK801 or NBQX and subjected to (**A**) TUNEL assay, (**B**) OCR analysis, and (**C**) calcium signal level analysis. The concentration of the drugs used: Dantrolene (50 μM, D50), Riluzole (1 μM, R1 or 0.5 μM, R0.5), MK801 (1 μM, MK1 or 0.5 μM, MK0.5) and NBOX 30, or 10 μM (N30, N10). Calcium signal data were compared among CTRL-, SCA2- and SCA3- iPSC-derived neurons treated with Dantrolene (50 μM), Riluzole (1 μM), MK801 (0.5 μM) or NBOX (30 μM) by measurement of Fluo-4 fluorescence intensity. All data represent the mean ± SD. *P < 0.05, **P < 0.01, ***P < 0.001. CTRL-1 & 2, SCA2-1 & 2 and SCA3-1 & 2 iPSC lines were used in the experiments.

Since our results demonstrated that glutamate caused pronounced cell death, mitochondrial dysfunction and increased cytosolic calcium in SCA-iPSC-derived neurons, we further tested whether blocking either glutamate receptors or calcium release could alleviate the observed SCA-associated pathological phenotypes. In order to achieve this goal, we assessed the effects of Dantrolene (a stabilizer of intracellular calcium ions), Riluzole (a mixed action antiglutamatergic agent), MK801 (an NMDA receptor antagonist), and NBQX (an AMPA receptor antagonist) on SCA-iPSC-derived neurons cultured in medium containing glutamate (Fig. S8). As measured by the TUNEL assay and OCR analysis, we found that Dantrolene (50 μM) treatment significantly reduced cell death and improved mitochondrial function in the SCA3-iPSC-derived neurons, but not in SCA2-iPSCs-derived neurons (Fig. 5A,B). On the other hand, Riluzole (1 μM treatment reduced cell death and improved the mitochondrial function in both SCA2- and SCA3-iPSC-derived neurons (Fig. 5A,B). Furthermore, MK801 (0.5 μM) improved mitochondrial function in neurons derived from both SCA-2 and SCA3-iPSCs, while NBQX (10 and 30 μM) did not significantly affect either cell death or mitochondrial function in neurons derived from both SCA-2 and SCA3-iPSCs (Fig. 5A,B). Next, we investigated the effects of the aforementioned drugs on intracellular calcium levels (Fig. 5C). By measuring cytosolic calcium with Fluo-4, we found that Dantrolene reduced calcium levels in SCA3-iPSC-derived neurons, but Riluzole reduced calcium levels in both SCA2- and SCA3-iPSC-derived neurons. MK801 and NBQX only reduced calcium levels in SCA3-iPSC-derived neurons, but not in SCA2-iPSC-derived neurons (Fig. 5C). These observations demonstrated that Riluzole (1 μM) improved all of the tested SCA phenotypes in both SCA2- and SCA3-iPSC-derived neurons, while Dantrolene (50 μM) exhibited more positive effects on SCA3-iPSC-derived neurons compared to those derived from SCA2 iPSCs. Overall the results suggest that drugs targeting either glutamate receptors or calcium flux may have beneficial effects on SCA-associated pathological phenotypes.

## Discussion

In this study, we describe the generation and neural differentiation of diseased SCA-iPSCs with mutations in *ATXN2* or *ATXN3*. Using SCA-iPSCs as an *in vitro* disease modelling platform, our studies revealed that the SCA-iPSC-derived neuronal populations exhibit vulnerability to glutamate. Glutamate has previously been shown to promote neuronal death in several neurodegenerative diseases, such as ALS, AD and HD^13^. Mechanistic analysis further suggested that long-term glutamate treatment on the SCA-iPSC-derived neurons impairs the expression of glutamate receptor genes and dysregulates intracellular calcium, which eventually leads to neuronal cell death. Alleviation of cell death and mitochondrial dysfunction by anti-glutamate drugs and a calcium stabilizer further supported the notion that glutamate receptor signaling plays a vital role in the development of SCA-relevant pathologic progression, and suggests that glutamate signaling may serve as a drug target for the development of new therapeutics for SCA treatment.

Neuronal cell death and polyQ aggregation are the phenotypic hallmarks of SCA2 and SCA3 disease. Although Koch *et al*. have demonstrated that ataxin-3 aggregates developed in SCA3-iPSC-derived neurons treated with glutamate (30 min, twice) by immunoblotting, no signs of macroinclusions or glutamate induced cell death were observed^9^. Consistent with these findings, our results showed that short-term glutamate treatment on the SCA-iPSC-derived neurons did not result in overt increases of neuronal cell death (Fig. S6)^9^. Nevertheless, we observed several SCA-relevant pathological phenotypes, including polyQ aggregation, cell death and mitochondrial dysfunction, in SCA-iPSC-derived neurons after prolonged glutamate treatment, suggesting that long-term glutamate exposure may promote the recapitulation of SCA-associated pathological phenotypes in the SCA-iPSC-derived neurons. Together, our findings demonstrate that the human SCA-iPSC model recapitulates the hallmarks of SCA pathogenesis and can be used to uncover further disease mechanisms governing the pathological progression of SCA2 and SCA3. Furthermore, the model can be developed into a platform for screening novel therapeutics.

Our results revealed that under long term glutamate culturing conditions, expression of several glutamate receptor subunit genes was reduced in SCA2- (GRIA4 and GRM3) and SCA3- (GRIA1, GRIA2, GRIA4 and GRM3) iPSC-derived neurons when compared to normal control-iPSC-derived neurons. Previous reports have indicated the subunit composition of AMPA receptors are dynamically remodeled during development and in response to the environmental changes, including neuronal activity, sensory experience and neuronal insult^14^. The reduction of GRIA2 expression has profound implications for synaptic efficacy and neuronal survival because this subunit limits calcium (and zinc) permeability^14–19^. Metabotropic glutamate receptors (mGluRs) of Group II (including GRM2 and GRM3) and Group III (including GRM4, GRM6, GRM7 and GRM8) are coupled to the inhibition of the cyclic AMP signaling and exert modulatory control on NMDA receptor activity^20^. Thus, downregulation of the receptors may enhance neurotoxicity caused by hyperactivity of the NMDA receptor. Several studies have suggested that altered expression of glutamate receptors would disturb calcium homeostasis, leading to cellular toxicity in neurodegenerative diseases. For example, in HD mouse models, mutant huntingtin was found to increase NMDA receptor activity and disturb calcium signaling^21,22^. In human HD-iPSC-derived striatal cultures, the upregulation of NMDA receptor subunit, GRIN2B, has also been shown to be associated with glutamate toxicity and calcium dyshomeostasis, upon acute BDNF withdrawal^23^. Glutamate receptor dysregulation has also been found in ALS, where protein expression of AMPA receptor subunit GRIA2 was found to be decreased. This decrease was suggested to contribute to disease pathology by increasing glutamate receptor mediated calcium influx^24,25^. Furthermore, downregulation of AMPA receptors was discovered in early Alzheimer’s disease (AD)^26^. Amyloid beta (Aβ) peptides were shown to reduce the level of GRIA1 and GRIA2 on the cell surface^27^. GRIA1 surface expression is potentially decreased via reduced Ca^2+^/Calmodulin-dependent protein kinase II, while GRIA2 decreases may arise from increased PKC-mediated phosphorylation^28,29^. In our results using SCA3-iPSC-derived neurons, we observed the downregulation of several AMPA receptor subunits as well as Ca^2+^/Calmodulin-dependent protein kinase IV. It is not clear whether the reduction of AMPA receptors in our results was associated with Ca^2+^/Calmodulin-dependent protein kinase. However, it is tempting to speculate that the downregulation of the glutamate receptor subunit genes in SCA2- and SCA3-iPSC-derived neurons might alter the receptor subunit composition, leading to changes in cell physiological properties, such as calcium homeostasis, and further causing neuronal cell death. Under glutamate-free culture conditions, cell death (with significant differences), lower basal OCR with blunted response to stressors, and increase of calcium influx were all observed in SCA3-iPSC-derived neurons. These phenomena correlated with the altered expression of several glutamate receptor subunits in SCA3-iPSC-derived neurons although the differences in gene expression were not statistically significant (Fig. 4Aa,Bb). It is not known whether the mutant *ATXN3* gene is directly involved in downregulating these glutamate receptor genes. Thus, we believe a more thorough investigation and screen of more SCA3-iPSC lines will be necessary to further investigate this finding.

Although polyQ aggregation is a characteristic feature for polyQ diseases, whether aggregates directly contribute to pathogenesis or represent a cellular strategy to inactivate toxic effects of expanded polyQ is still under debate^30^. In our study, IF staining showed that 1C2-positive aggregates were present in a few scattered SCA-iPSC-derived neurons. The quantification of IF staining results and a filter retardation assay demonstrated no statistically significant increase in aggregates when comparing SCA-iPSC-derived neurons with controls either in the presence or absence of glutamate (Figs 2Ac and S9). It has been suggested that the accumulation of aggregates is associated with a gradual decline in proteostasis and is promoted by aging in numerous neurodegenerative diseases^31^. Therefore, we suspect that our current *in vitro* SCA model might not age long enough to develop an appropriate level of macroaggregates to be detected in our current experiments. Studies using transgenic animals and cellular models have shown that the onset of neurological disease develops long before protein aggregates can be observed^32–35^. Several reports also indicated that the formation of oligomers or microaggregates, but not macroaggregates, is the primary factor that initiates the toxic effects^36–38^. In our results, we saw changes in glutamate receptor gene expression and pathology caused by long term culturing in a glutamate containing environment, however we did not observe the elevation of glutamate-induced macroaggregations. Our results showing pathological phenotype in the absence of overt macroaggregates are corroborated by these previous studies.

**Figure 6:**
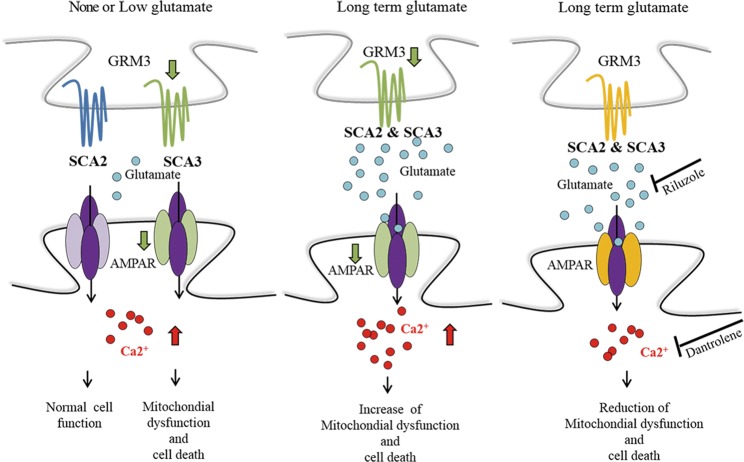
Proposed model for glutamate-mediated SCA pathogenesis. In SCA2, the expression of glutamate receptor related genes, such as GRM3 and GRIA4 are normal under low/none glutamate conditions, leading to normal cell function (*left panel*). However, after long term glutamate exposure, glutamate receptor related genes are downregulated. Subsequently, increased intracellular calcium impairs mitochondrial function and eventually causes cell death (*middle panel*). In SCA3, the expression of glutamate receptor related genes is reduced under low/none glutamate conditions (*left panel*), leading to impaired cellular function. Long term glutamate exposure worsens the expression and cellular function impairment (*middle panel*). Riluzole or Dantrolene can mitigate the cell dysfunction from glutamate induced cell death by anti-glutamate or calcium stabilizing actions.

To provide a proof of principle that SCA-iPSC-derived neurons may be used for drug testing, we explored the potential of various small molecules directed against glutamate or calcium to alleviate glutamate-induced pathological phenotypes in the SCA-iPSC-derived neurons. Our study reveals that Dantrolene and Riluzole significantly improve the pathological features of SCA2- and SCA3-iPSC-derived neurons, respectively, including cell survival, mitochondrial function, and calcium levels. Our results are in line with previous findings indicating that Dantrolene and Riluzole exert neuroprotective effect on various neurodegenerative diseases^39,40^. For example, Dantrolene can attenuate glutamate-induced cell death in a transgenic SCA2 mouse model through blockade of the ryanodine receptor and prevention of calcium ion release from the endoplasmic reticulum^39^. Riluzole, a drug used for ALS treatment, is also known to be effective against diverse forms of cerebellar ataxia. This drug exerts its neuroprotective effect by inhibiting glutamate release, inactivating voltage-dependent sodium channels, and noncompetitively blocking NMDA receptors^40,41^. In the case of MK801, a noncompetitive antagonist of the NMDA receptor, and NBQX, an AMPA/kainate antagonist, our results showed that the compounds can only improve some of the SCA-relevant phenotypes in the SCA2 and SCA3-iPSC-derived neurons. This result suggests that the blockage of glutamate, glutamate receptors, or downstream calcium signaling, by MK801 or NBQX, may help to alleviate certain pathological phenotypes in the SCA-iPSC-derived neurons and explain their neuroprotective effects in traumatic spinal cord injury and cerebral ischemia^42–44^. While our gene and protein expression data showed several AMPA subunit genes, especially GRIA4, were significantly downregulated in the SCA2- and SCA3-iPSC-derived neurons under long-term glutamate treatment, the drug against AMPA receptor (NBQX) did not show a strong protective effect. This observation supports the notion that the glutamate-mediated neurotoxicity may result from different types of glutamate receptors and their related signaling pathways. These results suggest that drugs with broader blocking effect on glutamate or associated downstream pathways might have better potential to protect against glutamate-mediated cell toxicity (Fig. 6).

## Materials and Methods

### Collection of parental cells and generation of patient-specific iPSCs

Dermal fibroblasts or PBMCs from four patients with either SCA2 or SCA3 diseases and one normal individual were obtained and used for iPSC generation (Supplementary Table 1). Two other control iPSC lines, CTRL-2 and CTRL-3, were generated from dermal fibroblasts^45^. Written informed consent was obtained in accordance with the protocol approved by the Research Ethics Committee (REC) of National Taiwan University Hospital and the Institutional Review Board (IRB) of Taipei Veterans General Hospital and Academia Sinica. All methods were performed in accordance with relevant guidelines and regulations. Derivation of iPSCs was accomplished either with retrovirus^46^, or non-integrated Sendai virus (CytoTune™-iPS 2.0 Sendai Reprogramming Kit, Life Technologies, Carlsbad, CA, USA) as the manufacturer described. Primers used to characterize exogenous and endogenous gene expression in newly generated iPSCs were previously described^46^. For all the experiments in this study, two clones each from SCA2 (SCA2-1 & 2) and SCA3 (SCA3-1 & 2) iPSC lines, and at least two control iPSC lines (CTRL-1 & 2) were used (Supplementary Table 1).

### Cell culture and differentiation

The maintenance and *in vitro* random differentiation of undifferentiated human iPSCs were performed as previously described^45^. For neuronal differentiation, iPSCs were dissociated with 1 mg/ml dispase (Sigma), and then left to form embryoid bodies (EBs) in ultra-low attachment dishes (Corning, Corning, NY, USA) for 4 d, in 20% knockout serum replacement media, consisting of DMEM/F12, 20% knockout serum replacement, 1% NEAA, and 2 mM L-glutamine with 100 mM 2-mercaptoethanol, and containing 10 μM SB431542 and 100 nM LDN193189. EBs were then switched to N2 media, consisting of NeuroBasal medium, 1X N2 supplement, 1 mM sodium pyruvate, 2 mM L-glutamine, and 20 ng/mL bFGF (all purchased from Thermo Fisher Scientific, Waltham, MA, USA), for another 2 d. Next, EBs were plated on Matrigel (BD Biosciences, Franklin Lakes, NJ, USA) coated tissue culture dishes (Corning) in N2 media until neural rosettes appeared. Rosettes were manually picked and cultured as spherical aggregates in N2 medium for 4 to 6 d. Afterward, neurospheres were attached to Matrigel-coated dishes in N2 medium until day 30, then switch to B27 media (NeuroBasal medium, 1X B27 supplement, 1% NEAA, 1 mm sodium pyruvate, 2 mM L-glutamine, 20 ng/ml bFGF and 300 ng/ml cAMP) for further neuronal maturation.

For our standard neuronal culture procedure, B27 media was usually supplemented with 1% NEAA (100 μM glutamate final concentration). However, in order to analyze the effect of glutamate on disease phenotype, media were switched to B27 medium without NEAA and then supplemented either with or without additional glutamate (100 μM, Sigma). Media was changed every 2 d. For subculture of neural progenitors, the iPSC-derived neural rosettes were picked and cut into small pieces (1–2 mm in diameter) using a needle, every 10 to 12 d, and then plated onto Matrigel-coated dishes. For all the assays performed in this experiment, iPSC-derived neurons were dissociated between day 100 and 120 with Accutase (Thermo Fisher Scientific) and counted by the Scepter™ 2.0 Cell Counter (Merck Millipore, Billerica, MA, USA) before plating. Cells were assayed the next day.

### Drug treatments

SB431542, Dantrolene and Riluzole were purchased from Sigma Aldrich (St Louis, MO, USA). LDN193189 was purchased from STEMGENT (San Diego, CA, USA). MK801, NBQX and AMPA were purchased from Tocris Bioscience (Bristol, UK). For anti-glutamate drug treatment, 4 month differentiated iPSC-derived neurons cultured with glutamate were used, and drugs were added on the day after plating cells in media without glutamate. Cells were pre-incubated with drugs in Neurobasal medium without glutamate for 2 hr, then treated with drug overnight in B27 medium with glutamate. Cell death, as measured by the TUNEL assay, and visual inspection were used to decide the best drug working concentrations for disease-derived neurons. Dantrolene was tested at 50, 25 and 10 μM; Riluzole and MK801were tested at 5, 3, 1 and 0.5 μM; and NBQX was tested at 30, 10 and 5 μM. Riluzole and MK801 at 3 to 5 μM were found to cause unhealthy cell morphology in both control and disease-derived neurons after overnight treatment. Therefore, the assay results at these doses were excluded.

### Cell death: TUNEL assay

Neurons derived from iPSCs were dissociated into single cells, seeded onto Matrigel-coated coverslips, and then cultured for 2 d before experimentation. The TUNEL assay was performed using the DeadEnd™ Fluorometric TUNEL System (Promega, Wisconsin, USA), according to the manufacturer’s instructions. The percentage of TUNEL-positive cells was calculated from the total number of TUJ1^+^ cells by MetaMorph Microscopy Automation & Image Analysis Software (Molecular Devices, Sunnyvale, CA, USA).

### Seahorse XF-24 metabolic flux analysis

Neurons derived from iPSCs were cultured on Matigel-coated Seahorse XF-24 (Seahorse Bioscience, North Billerica, MA, USA) plates at a density of 1 × 10^5^ cells per well and assayed the next day. The measurement of intact cellular respiration was performed according to the manufacturer’s protocol. The compounds used in the mitochondrial stress test were oligomycin (2 μM), FCCP (2 μM), and antimycin (2 μM). After the assays, protein content of each well was measured to confirm equal cell number per well.

### Measurement of cytoplasmic calcium with Fluro-4

IPSC-derived neurons were loaded with 3 μM Fluo-4 AM (Thermo Fisher Scientific) in HEPES-buffered physiological salt solution for 1 h at 25 °C. The procedure was performed as previously described^47^. Cells were visualized using a Leica inverted microscope and living cell system (Leica Microsystems, Wetzlar, Germany). Images were analyzed by MetaMorph Microscopy Automation & Image Analysis Software (Molecular Devices).

### RNA extraction and quantitative polymerase chain reaction

Total RNA was extracted with RNeasy Mini Kit (Qiagen, Germantown, MD, USA) and treated with DNase I (Qiagen). RNA was converted to cDNA using the High Capacity cDNA Reverse Transcriptase Kit (Life Technologies). Polymerase chain reaction (PCR) was performed using the Platinum Taq DNA polymerase kit (Life Technologies) according to the manufacturer’s instructions. Reverse transcriptase quantitative PCR (RT-qPCR) was performed with the SYBR^®^ Select Master Mix (Life Technologies), and analyzed with the 7900 real-time PCR system (Life Technologies). Gene specific primers are listed in Table S2. Results were normalized to GAPDH, and relative expression was calculated according to the ΔΔC_t_ method.

### Genomic PCR and repeat length analysis

Genomic DNA was extracted using DNeasy Blood & Tissue Kit (Qiagen). The primer sets used for SCA2 were: SCA2-A (5′-GGGCCCTCACCATGTCG-3′) and SCA2-B (5′-CGGGCTTGCGGACATTGG-3′). For SCA3, MJD52 (5′-CCAGTGACTACTTTGATTCG-3′) and MJD25 (3′-AAGTGTAGGTACACTTTCCGGT-5′) were used. PCR conditions were as previously described^48,49^. The PCR fragments were extracted from agarose gels and sequenced to determine the CAG repeat length.

### Immunofluorescence staining

Immunofluorescence (IF) staining was performed as previously described^45^. Primary antibodies used in this study were: OCT4 (1:200), SSEA4 (1:200), TRA-1-60 (1:200), MAP2 (1:500), TUJ1(1:500), ZIC1 (1:500), ZIC3 (1:500), ATH1 (1:500), LHX5(1:500), GAD67 (1:200), Anti-polyQ-expansion disease marker (1C2) (1:10000) (Millipore, Temecula, CA, USA), PAX6 (1:50) (DSHB, Iowa City, IA, USA), AFP (1:500) (Dako, Glostrup, Denmark), SOX17 (1:200) (R&D) (1:500), GATA4 (1:200) (Santa Cruz, Dallas, Texas, USA), α-SMA (1:500) (Sigma) (1:800) (Abcam, Cambridge, UK), and ZIC2 (1:200) (Aviva Systems Biology, San Diego, CA, USA). The following secondary antibodies were used: goat anti-mouse, -rabbit Cy3 (1:500) (Jackson ImmunoResearch laboratories, West Grove, PA, USA), and goat anti-mouse, -rabbit 488 (1:200) (Life Technologies).

### Electron microscopy analysis, teratoma generation, chromosome karyotype analysis, Western blot and filter retardation assay

Methods are described in the supplemental Materials and Methods.

### Microarray and gene pathway analysis

Two μg of total RNA from CTRL-1, CTRL-2, CTRL-3, SCA2-1, SCA2- 2, SCA3-1, and SCA3-2 iPSC-derived neurons at day 100–120 of neural differentiation were used to generate biotin-labeled cRNA probes, which were hybridized to the Affymetrix Human Genome U133 plus 2.0 array (Affymetrix, Santa Clara, CA, USA) by the Affymetrix gene expression service laboratory at Academia Sinica (GRC, Academia Sinica, Taipei, Taiwan). Chips were scanned with an Affymetrix GeneChip Scanner 7G, and data were analyzed with GeneSpring X software (Agilent, Santa Clara, CA, USA). Raw data were normalized independently for each cell line using the Robust Multichip Average method. By comparing the gene expression from the data set of control and SCA2 or SCA3 iPSC lines, expression changes with a fold change >2 and an adjusted *P* < 0.05 were considered to be significantly different, and analyzed further for their biological function in the canonical networks with the Ingenuity Pathway Analysis (IPA) system (Qiagen; www.ingenuity.com). For all analyses, Fisher’s exact test was used to calculate a *P*-value determining the probability that the association between the significantly different genes in the data set and the canonical pathway was due to chance alone. The microarray data have been deposited in the Gene Expression Omnibus (GEO; www.ncbi.nlm.nih.gov/geo, Accession No. GSE96826).

### Statistical analyses

All experiments were performed with at least three biological replicates. Results are shown as the mean ± SD. Student’s *t*-test was used to examine the significance of differences between two groups; comparisons among three groups were made by one-way ANOVA with a Tukey’s *post hoc* test. *P* < 0.05 was considered to be statistically significant.

## Supplementary information

Supplementary information

## Data Availability

All data generated and analyzed during this study are included in this article (and its Supplementary Information files).
