# Supplementary material for: Modeling spinocerebellar ataxias 2 and 3 with iPSCs reveals a role for glutamate in disease pathology
Source: Sci Rep. 2019 Feb 4;9:1166. doi: 10.1038/s41598-018-37774-2 (PMC6361947; doi:10.1038/s41598-018-37774-2)
Supplement: Supplementary file 1 — Supplementary information [file 41598_2018_37774_MOESM1_ESM.pdf]

Modeling spinocerebellar ataxias 2 and 3 with iPSCs reveals a major role of glutamate in disease pathology

Ching-Yu Chuang[1,2], Chih-Chao Yang[3], Bing-Wen Soong[4], Chun-Ying Yu[2], Shu-Hwa Chen[5], Hsiang-Po Huang[6], Hung-Chih Kuo[1,2,6, 7*]

[1]Genomics Research Center, Academia Sinica, Taipei, Taiwan

[2]Institute of Cellular and Organismic Biology, Academia Sinica, Taipei, Taiwan

[3]Departments of Neurology, National Taiwan University Hospital, Taipei, Taiwan

[4]Departments of Neurology, National Yang-Ming University Faculty of Medicine and Taipei Veterans General Hospital, Taipei, Taiwan

[5]Lab of Systems and Network Biology, Institute of Information Science, Academia Sinica, Taipei, Taiwan

[6]Graduate Institute of Medical Genomics and Proteomics, College of Medicine, National Taiwan University, Taipei, Taiwan

[7]Graduate Institute of Clinical Medicine, Taipei Medical University, Taipei, Taiwan

*To whom correspondence should be addressed:

Hung-Chih Kuo

Institute of Cellular and Organismic Biology, Academia Sinica, 128 Academia Road, Sec. 2, Nankang Dist., Taipei 115, Taiwan

Phone: +886-2-2789-9580 ext. 201. Fax: +886-2-2789-9587

Email: kuohuch@gate.sinica.edu.tw

Supplementary table 1:
List of iPSC lines generated in this study

	Age (at time of sample collection)	Sex	Repeat length	iPSC clones used in this study	Method of iPSC generation & parental cell type
CTRL-1	42	Female	_	iPBMC-B13	Sendai virus (PBMC)
CTRL-2	36	Female	_	iNHF-5 (Ref 45)	Retrovirus (dermal fibroblast)
CTRL-3	newborn	Male	_	iHFF-1(Ref 45)	Sendai virus (foreskin fibroblast)
SCA2-1	57	Male	35/22	iSCA2-17<br>iSCA2-28	Retrovirus (dermal fibroblast)
SCA2-2	36	Female	44/22	iSCA2 E209-6<br>iSCA2 E209-9	Sendai virus (PBMC)
SCA3-1	38	Female	70/37	iSCA3-1<br>iSCA3-36	Retrovirus (dermal fibroblast)
SCA3-2	39	Female	73/14	iSCA3 E149-10<br>iSCA3 E149-12	Sendai virus (dermal fibroblast)

Supplementary table S2:

Primer sets used for Quantitative RT-PCR

Primer Name	Sequence (5′ to 3′)
GRIA1-F	AATGTGGCAGGCGTGTTCTAC
GRIA1-R	AACTCGATTAAGGCAACCAGCAT
GRIA2-F	TGGTGGTTCTTTACCCTGATCAT
GRIA2-R	CTCTACAGTCAGGAAGGCAGCTAA
GRIA4-F	GTGCCCTTTTTCATGTCTTTCC
GRIA4-R	CAGACGAGTACACAGGCAATTACA
GRM3-F	GCTCCATTCAACCCAAATAAAGA
GRM3-R	CCCATTCCATCTCCAAAAGTGT
SLC17A6-F	GAGTGCATTATGGATGTGTCATCTT
SLC17A6-R	GGGTAGGTCACACCCTCAACA
GAPDH-F	CGGGAAACTGTGGCGTGATG
GAPDH-R	TG TGGAGGAGTGGGTGTCGCTGTT

Figure S1

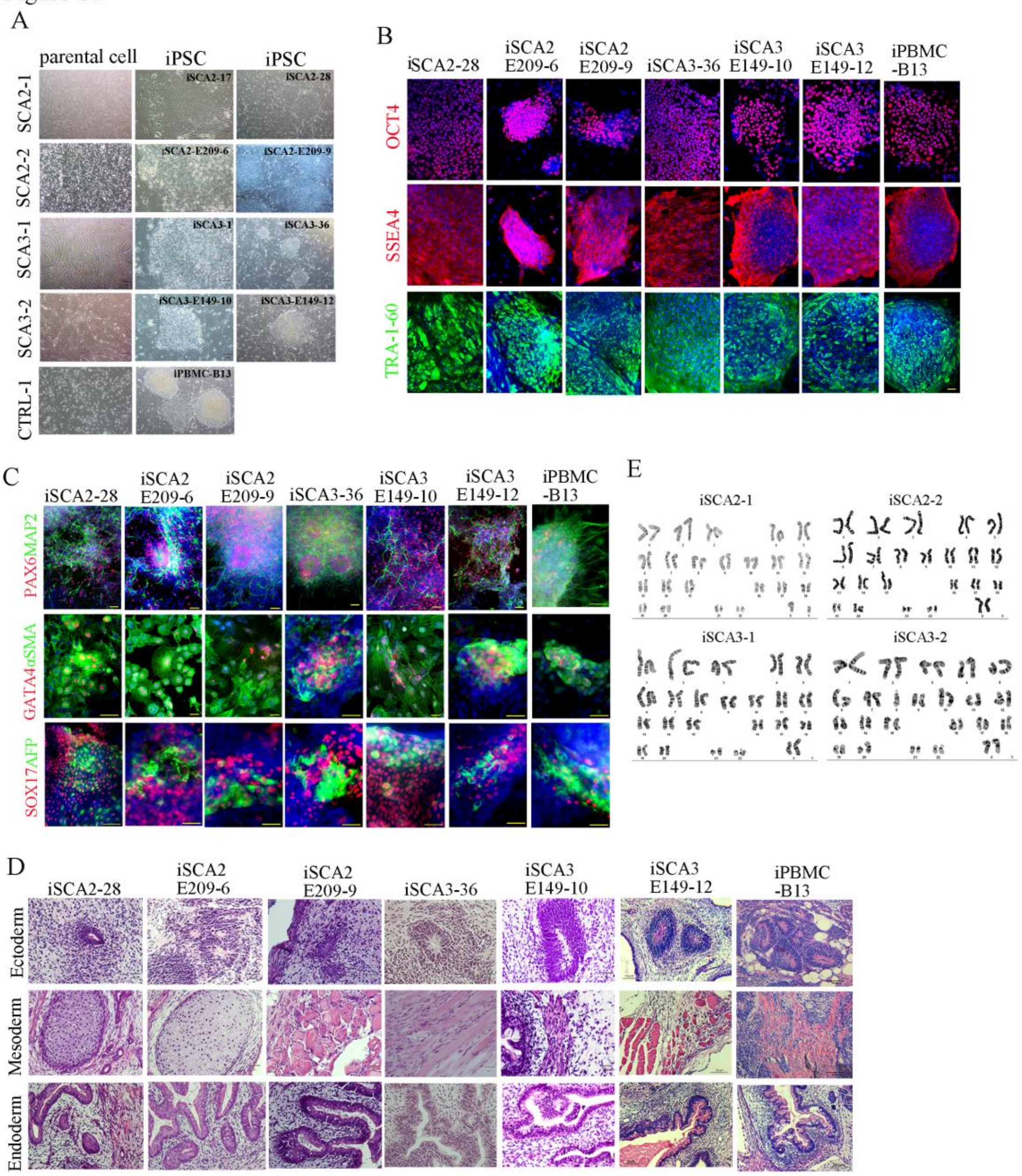

Figure S1: Characterization of SCA- and control-iPSCs. (A) Phase contrast images of parental cells and reprogrammed iPSC colonies. Scale bars, iPSCs, SCA2-2, CTRL-1 PBMC and SCA3-1 fibroblast: 100 µm; SCA2-1 and SCA3-2 fibroblasts: 300 µm. (B) Immunofluorescence staining of iPSCs with antibodies against pluripotency-associated antigens OCT4, SSEA4 and TRA-1-60. Scale bar: 50 µm. (C) Immunofluorescence staining of differentiated iPSC with antigens against lineage-specific antigens PAX6 and MAP2 (ectoderm), GATA4 and α-SMA (mesoderm), and SOX17 and AFP (endoderm). Scale bar: 50 µm. (D) Hematoxylin and eosin staining of teratomas derived from SCA-2-2 and SCA3-2 iPSCs. Scale bars: 30 µm. (E) Chromosome karyotypes of iPSC lines.

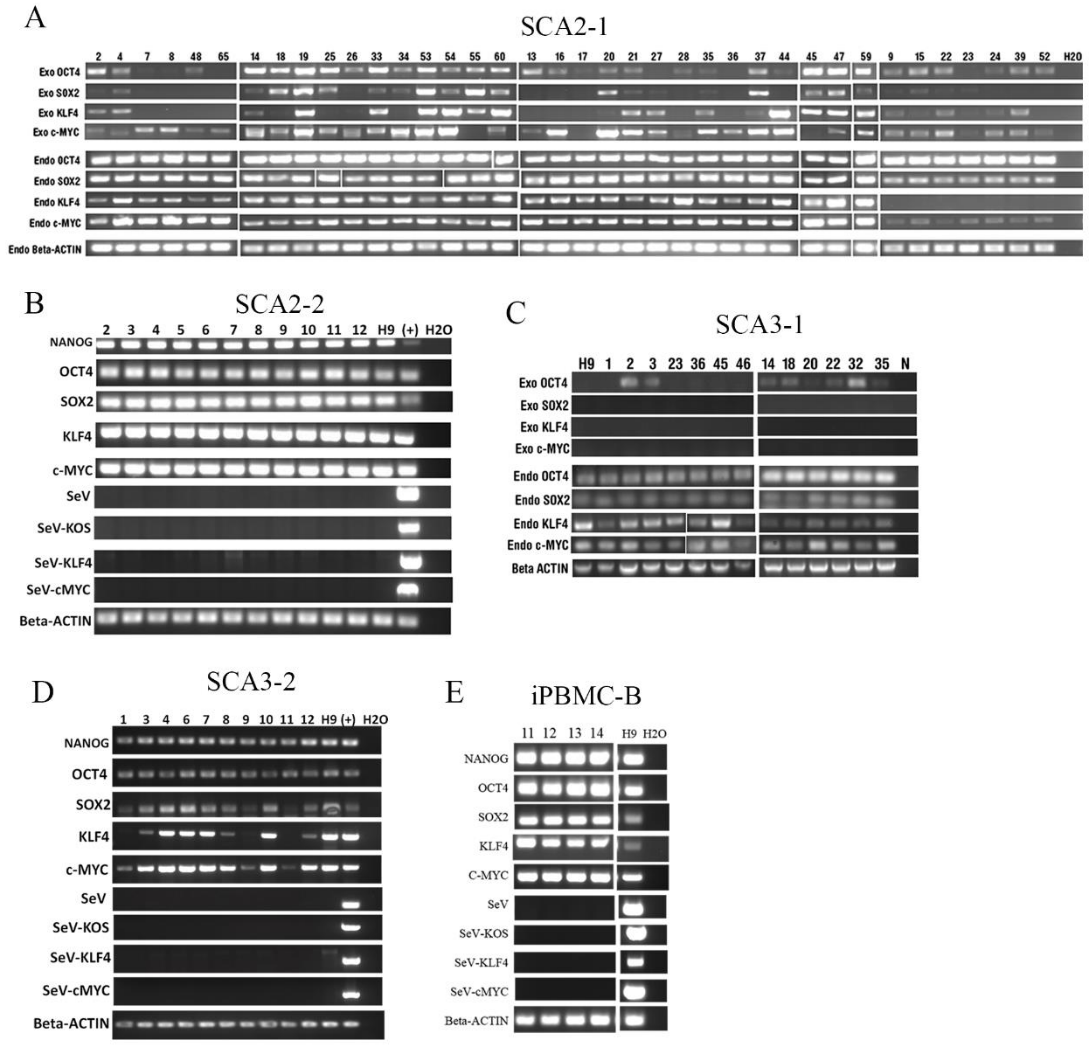

Figure S2: Genetic analysis of iPSCs. RT-PCR was used to confirm the inactivation of exogenous retro or Sendai viral transgenes and expression of endogenous pluripotency-associated genes as indicated.

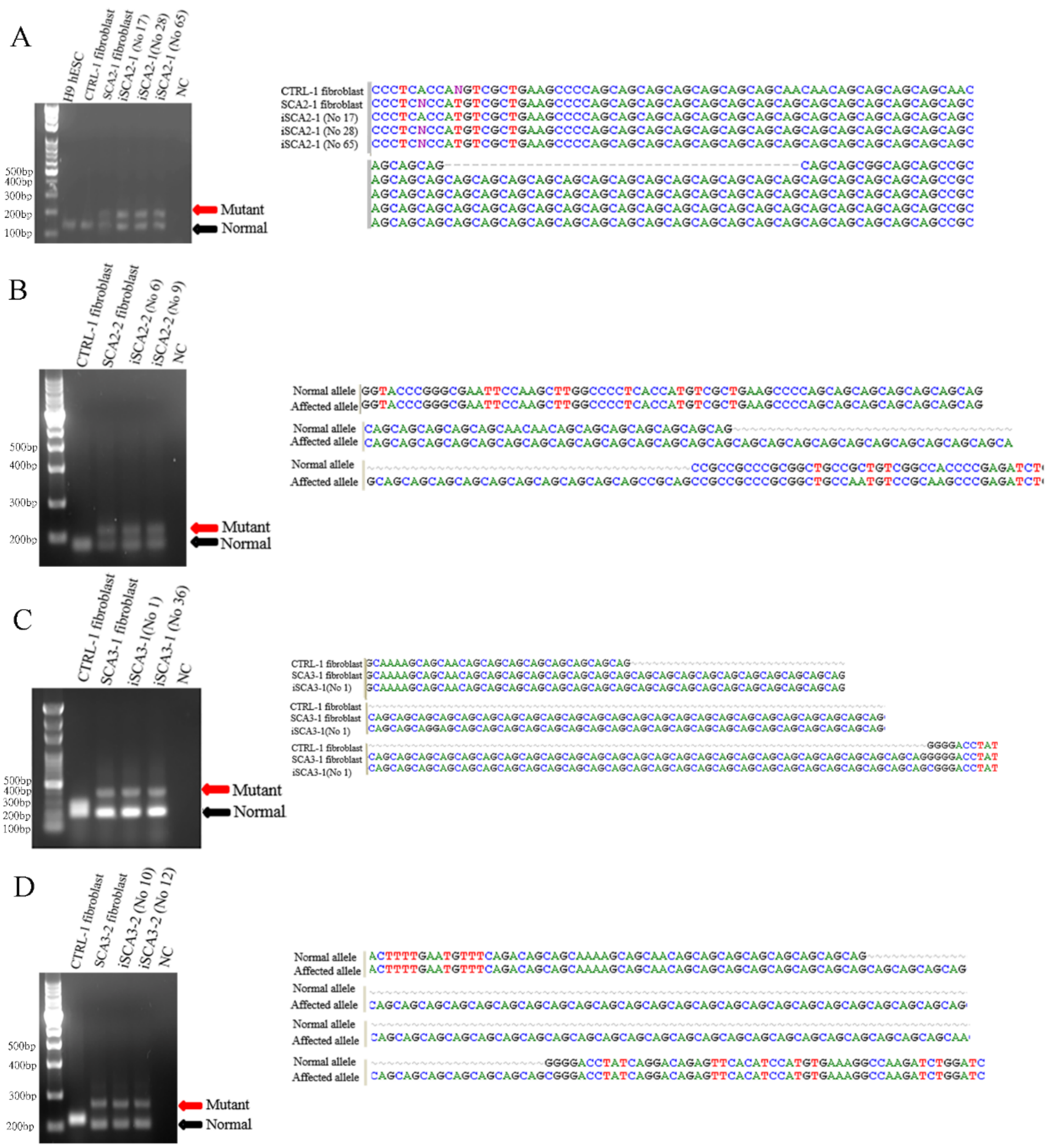

Figure S3: PCR and sequencing analysis confirmed CAG expansions in SCA2- and 3-iPSCs. Genomic DNA isolated from normal control fibroblast and (A) SCA 2-1-, (B) SCA2-2-, (C) SCA3-1- and (D) SCA3-2-iPSCs were subject to PCR and sequencing analysis. The transcripts of both normal and expanded alleles were expressed in the disease-iPSC clones. Sequencing results also confirmed the expanded CAG repeats in SCA-iPSCs.

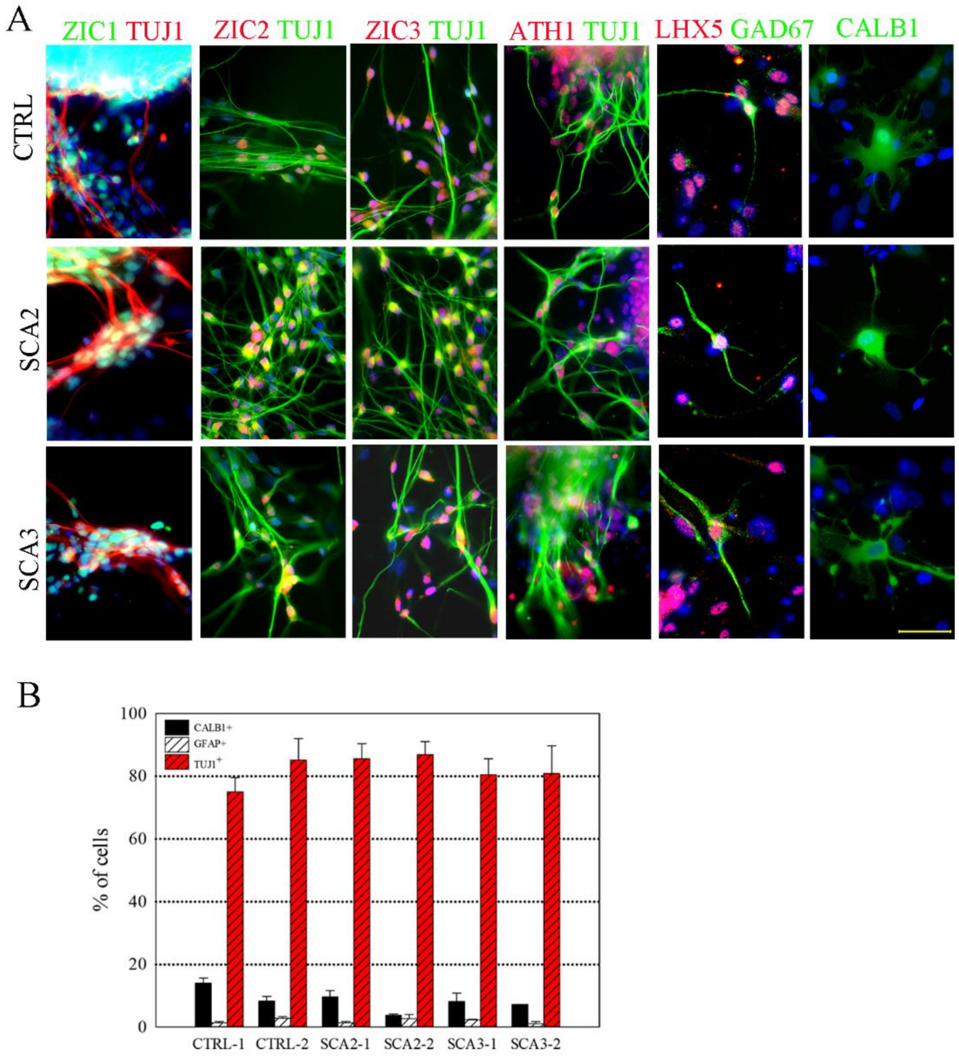

Figure S4: Immunostaining of (A) cerebellar cell markers in representative SCA-iPSC derivative cultures and (B) quantification of CALB1, GFAP and TUJ1 positive cells at day 90 of neuronal differentiation. Nuclei were stained with DAPI. Scale bar: 50 μm.

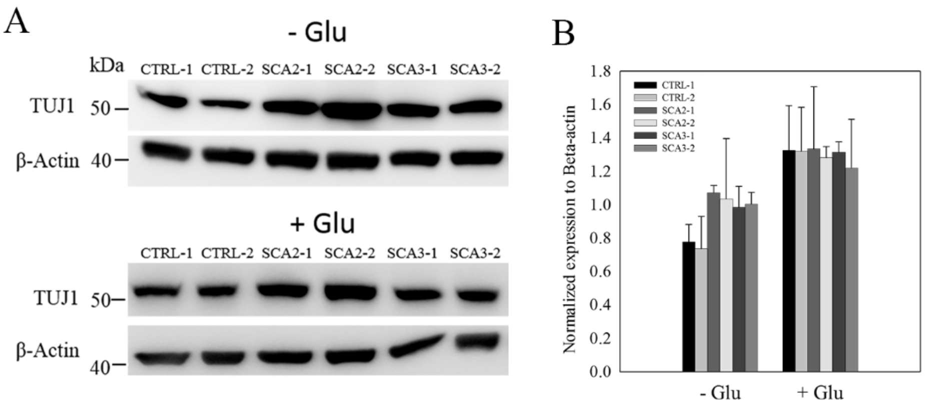

Figure S5: (A) The expression and (B) quantification of neuronal marker (TUJ1) in control- and SCA-iPSC-derived neurons. All data represent the mean ± SD. – Glu: culture medium without glutamate. + Glu: culture medium with glutamate.

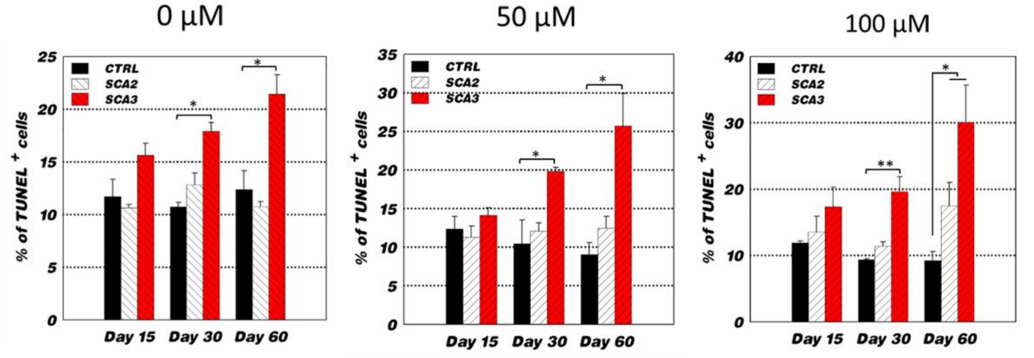

Figure S6: Dose and time course of glutamate treatment on SCA-iPSC-derived neuronal cultures. SCA-iPSC-derived neuronal populations were treated with 0, 50 or 100 μM glutamate for 15, 30 and 60 d. The TUNEL assay was performed on CTRL-3 (iHFF-1), SCA2-1, and SCA3-1-iPSC-derived neurons at neural differentiation day 120. The percentage of TUNEL-positive cells was calculated from the total number of TUJ1$^+$ cells. All values are means ± SD. * $P < 0.05$, ** $P < 0.01$.

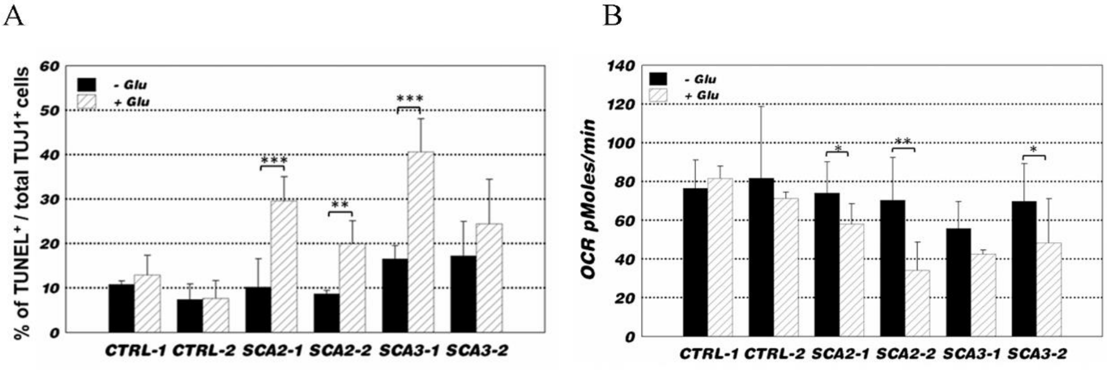

Figure S7: Comparison of cell death and mitochondrial respiration rate between glutamate treated and no glutamate-treated SCA-iPSC-derived neuronal populations. (A) TUNEL assay (B) Basal oxygen consumption rate (OCR). All values are means ± SD. * $P < 0.05$, ** $P < 0.01$. *** $P < 0.001$. – Glu: culture medium without glutamate. + Glu: culture medium with glutamate.

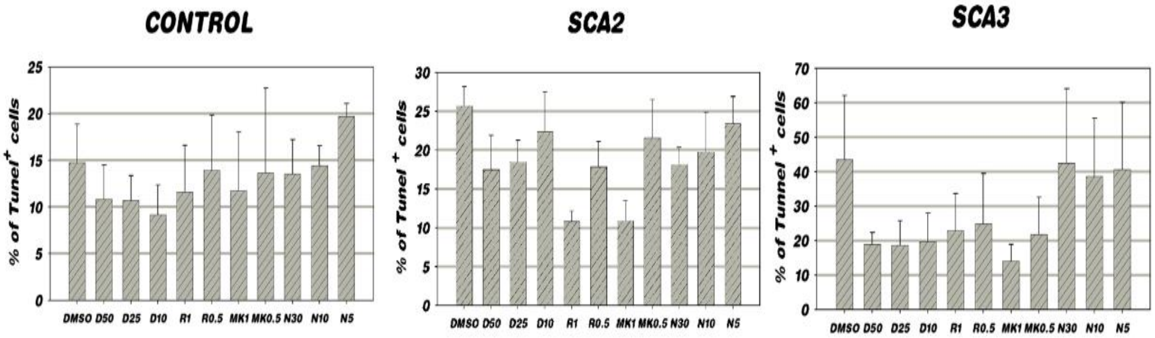

Figure S8: Cell death analysis by TUNEL assay on iPSC-derived neuronal populations treated with various drugs (as indicated). Dantrolene 50, 25, 10 µM (D50, D25, D10), Riluzole 1, 0.5µM (R1, R0.5), MK801 1, 0.5µM (MK1, MK0.5) and NBOX 30, 10, 5µM (N30, N10, N5). All data represent the mean ± SD.

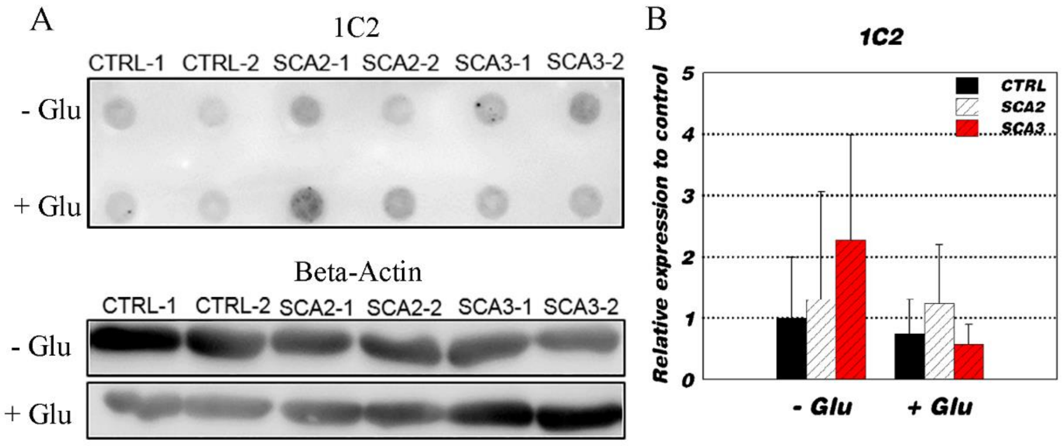

Figure S9: Detecting polyQ aggregation in the SCA-iPSC-derived neuronal populations. (A) Filter retardation assay of CTRL-, SCA2- and SCA3-iPSC-derived neuronal populations. The aggregates that were trapped on the filter were detected by 1C2 antibody. (B) Quantitative analysis of the blot shown in panel A. The 1C2 intensity was first normalized to β-ACTIN, then fold change compared to control samples was calculated. All data represent the mean ± SD. – Glu: culture medium without glutamate. + Glu: culture medium with glutamate.

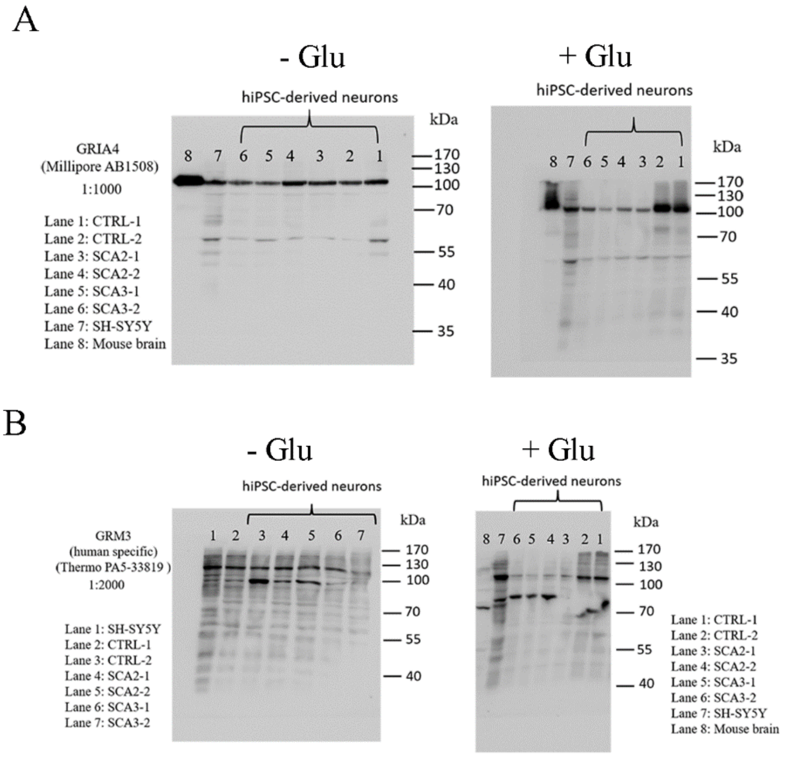

Figure S10: The full-length western blots showed in figure 4B. Antibodies against (A) GRIA4 or (B) GRM3 proteins.

Supplemental Materials and Methods:

Electron microscopy analysis

IPSC-derived neurons were cultured on Matrigel-coated ACLAR film for 2 d and fixed in 2% paraformaldehyde and 2.5% glutaraldehyde (Electron MicroscopySciences, Hatfield, PA, USA) in 0.1 M Cacodylate buffer (pH 7.4) overnight at 4 ℃. After fixation, samples were sent for the sequential procedures and analysis by the electron microscope core facility of Academia Sinica (IBMS, Academia Sinica, Taipei, Taiwan). The sections were examined under a Hitachi 7000 electron microscope (Hitachi, Tokyo, Japan).

Teratoma generation

All animal experiments were approved by the Institutional Animal Care and Use Committee of Academia Sinica. The detailed protocol for teratoma generation was performed as described previously [45]. Hematoxylin and eosin staining was performed using an Autostainer XL Leica ST5010 (Leica Microsystem).

Western blot and filter retardation assay

Cells were lysed in RIPA buffer (50 mM Tris-HCl buffer, pH 8.0, 150 mM NaCl, 1% NP-40, 0.5% sodium deoxycholate, 0.1% SDS, and protease inhibitor cocktail; Roche Diagnostics, Basel, Switzerland), and protein concentration was determined by the Pierce BCA Protein Assay Kit (Thermo Scientific, Waltham, MA, USA). For western blot analysis, 20 μg of sample protein was subjected to SDS-PAGE before being electroblotted onto a nitrocellulose membrane (Millipore). For filter retardation assay, cell lysate samples (10 or 30 μg/spot, diluted in PBS with 2% SDS) were loaded on a cellulose acetate membrane (0.2 μm, Advantec MFS, Inc., Dublin, CA, USA). Membranes were blocked in 5% skim milk in TBS, then sequentially probed with

primary and secondary antibody. SuperSignal West Femoto Maximum Sensitivity substrate (Thermo Scientific) was used to generate the signal. Images were acquired on a LAS-4000 mini device (FUJI Photo Film, Tokyo, Japan). The quantification of the protein bands or spots was accomplished using Image J (NCBI). Each set of experiments was repeated at least three times to confirm the results. The antibodies used were: Anti-polyQ-expansion disease marker (1C2, 1:500), β-actin (1:100,000), GRIA4 (1:1000) (Millipore), GRM3 (1:2000, human specific) (Thermo Scientific), goat anti-rabbit (1:10,000) and rabbit anti-mouse (1:50,000) HRP-conjugated antibodies (Sigma).
